# Datasets of chemical compounds in three different species of aquilaria using GC-MS coupled with GC-FID analysis

**DOI:** 10.1016/j.dib.2024.110209

**Published:** 2024-02-17

**Authors:** Zakiah Mohd Yusoff, Nurlaila Ismail

**Affiliations:** a College of Engineering, Universiti Teknologi MARA, 81750 Pasir Gudang, Johor, Malaysia; b School of Electrical Engineering, College of Engineering, Universiti Teknologi MARA, 40450, Shah Alam, Selangor, Malaysia

**Keywords:** Aquilaria crassna, Aquilaria maleccensis, Aquilaria subintegra, Agarwood oil, GC-MS, GC-FID chemical compounds

## Abstract

Aquilaria oil, specifically agarwood oil, is esteemed for its unique fragrance and potential therapeutic qualities, primarily attributed to the presence of significant chemical compounds. These compounds play a vital role in shaping the quality and attributes of Aquilaria oil. The distinct aroma, characterized by intricate, woody, and multifaceted notes, originates directly from specific sesquiterpenes, with notable contributors like agarospirol defining this aromatic profile. The richness and complexity of the oil's scent are closely linked to the concentration and variety of noteworthy compounds within it. Oils containing a diverse range of sesquiterpenes are often considered superior, providing a more refined olfactory experience. This dataset presents a statistical analysis of the chemical compounds present in agarwood oil obtained through the hydrodistillation method from three distinct Aquilaria (A.) species: A. crassna, A. malaccensis, and A. subintegra. The analysis of these chemical compounds utilized Gas Chromatography-Mass Spectrometer (GC-MS) coupled with Gas Chromatography – Flame Ionization Detector (GC-FID). This study's data is crucial for highlighting compounds that contribute to the significance of agarwood oil as a valuable and versatile natural resource. This significance is emphasized by the oil's diverse applications and distinctive chemical composition.

Specifications Table

**Table:** 

Subject	Chemistry
Specific subject area	Chemical composition
Data format	Raw, Analysed
Type of data	Excel Table
Data collection	In this study, two samples from each species, designated as ACS1 and ACS2 for A. crassna, AMS1 and AMS2 for A. malaccensis, and ASS1 and ASS2 for A. subintegra, respectively, were utilized. These samples were sourced from Malaysia, each having a volume of 5ml. The research incorporated an agarwood chip obtained from a natural forest in Rompin, Pahang, with extraction conducted using the hydrodistillation (HD) method. Various parameters were applied during each extraction to determine the optimal conditions for agarwood oil extraction. Prior to extraction, the ground agarwood chip underwent sequential soaking in water for several days to facilitate the breakdown of parenchymatous and oil glands. Subsequently, the samples were diluted in analytical-grade dichloromethane (DCM) for determination using gas chromatography-mass spectrometer (GC-MS) and gas chromatography system (GC-FID).
Data source location	Agarwood oil obtained from three Aquilaria species, namely A. crassna, A. malaccensis, and A. subintegra originating in Malaysia. The study employs an agarwood chip acquired from a natural forest situated in Rompin, Pahang.
Data accessibility	Data are with the article. Repository name: Mendeley Data Data identification number: 10.17632/9kpt7k5dd3.1 Direct URL to data: https://data.mendeley.com/datasets/9kpt7k5dd3/1

## 1 Value of the Data

• The dataset provides a statistical analysis of the chemical compounds present in agarwood oil from three specific Aquilaria species. This analysis contributes to the scientific understanding of the composition of agarwood oil, offering insights into its chemical makeup.
• By examining A. crassna, A. malaccensis, and A. subintegra, the data allow for a comparative analysis of chemical compositions among different Aquilaria species. This information is crucial for understanding the variations and similarities in agarwood oil profiles and valuable for industries involved in agarwood production, ensuring the quality and authenticity of their products.
• These datasets are valuable for researchers and scientists in various fields, including pharmacy, chemistry, biology, and environmental science. The dataset can be used to study the chemical properties and potential medicinal applications of these species, as well as to investigate their environmental impacts and conservation efforts.

## 2 Background

Aquilaria is recognized by distinct names across various countries. In Malaysia, it is referred to as *Gaharu,* while in China, it goes by the name *Chexiang*. Alternative names for Aquilaria also known as agarwood include *Kalambak, Kanankoh, Agalloch, Jinkoh, Eaglewood*, and *Aloeswood*. This non-timber forest product is in high demand and holds significant value, primarily attributed to its pivotal role as a key ingredient in herbal and medicinal production, as well as in the creation of fragrant goods. Furthermore, agarwood plays an essential role in various religious and cultural ceremonies and festivals. The chemical composition of agarwood varies depending on the species and environmental conditions, making it a complex and fascinating subject for scientific research [1,2]. In recent years, there has been increasing interest in the study of agarwood due to its potential medicinal applications and environmental significance.

## 3 Data Description

The datasets presented in this article consist of detailed chemical analyses of three different species of Aquilaria using GC-MS and GC-FID techniques. The datasets consist of two samples from each of the three species of Aquilaria, specifically A. crassna (designated as ACS1 and ACS2), A. malaccensis (designated as AMS1 and AMS2), and A. subintegra (designated as ASS1 and ASS2). The datasets include information on the Retention times, Tr (min), Peak Area (%), identified compounds, molecular formulas, molecular weights, identification modes, and similar chemical compounds found in every species of Aquilaria, were meticulously tabulated in MS Excel. Concurrently, the chemical analyses collectively unveil that agarwood oil is predominantly composed of a mixture of compounds. These compounds can be categorized into four distinct groups: carboxylic acid, other compounds, sesquiterpene, and sesquiterpenoid. This comprehensive dataset facilitates a thorough understanding of the chemical profiles and analytical nuances across the different Aquilaria species, enhancing the robustness and interpretability of the datasets.

## 4 Experimental Design, Materials and Methods

The extraction process was conducted by the BioAromatic Research Centre of Excellence (BARCE) at Universiti Malaysia Pahang (UMP). Various extraction parameters were applied to optimize the conditions for obtaining the agarwood oil extract. Preceding the extraction process, the ground agarwood chip underwent sequential water soaking for several days to facilitate the breakdown of parenchymatous and oil glands. Subsequently, the oil extraction was performed over a period of 3 to 5 days utilizing the hydro distillation process*.*

For GC-MS and GC-FID analyses, the samples were diluted in dichloromethane (DCM) of analytical grade. Identification in GC-MS involved a comparison of the mass spectrum generated from sample analysis with the National Institute of Standards and Technology (NIST) library, requiring a minimum similarity of ≥80%. GC-FID identification relied on linear retention indices, determined relative to the retention times on a DBI column of a homologous series of C [3].

The GC-MS system, an Agilent 7890B GC System coupled with an Agilent 5977A MSD, featured an inlet temperature set at 250°C, employing an Agilent DB-1ms column (30 m × 250 µm × 0.25 µm) with a helium flow rate of 1.0 mL/min. The oven program initiated at 80°C, with a 3°C/min increase until reaching 250°C, held for 3 minutes. Additionally, the auxiliary heater was set at 260°C, the MS source at 230°C, and the MS quad at 150°C, utilizing Electron Impact (EI) mode with an energy of 70 electron volts (eV) [4].

In contrast, the GC-FID system used an Agilent 7890B GC System with an inlet temperature of 250°C, the same Agilent DB-1ms column, and a helium flow rate of 1.0 mL/min. The oven program mirrored that of the GC-MS system, starting at 80°C, increasing by 3°C/min until 250°C, and maintaining this temperature for 3 minutes. Unlike the GC-MS system, the GC-FID system did not employ an auxiliary heater; instead, the FID detector operated at a temperature of 250°C.

For chemical component identification, mass spectral libraries (HPCH2205.L, Wiley7Nist05.L, and NIST05a.L) were consulted, and the findings were expressed in terms of peak areas using peak counts [2,5].

## Limitations

Not applicable.

## Ethics Statement

The authors have read and follow the ethical requirements for publication in Data in Brief and confirming that the current work does not involve human subjects, animal experiments, or any data collected from social media platforms.

## CRediT authorship contribution statement

**Zakiah Mohd Yusoff:** Conceptualization, Writing – review & editing, Funding acquisition. **Nurlaila Ismail:** Resources, Validation, Methodology, Investigation, Formal analysis.

## Data Availability

Datasets of Chemical Compounds in Three Different Species of Aquilaria using GC-MS coupled with GC-FID Analysis (Original data) (Mendeley Data).
